# Phylogeography and genetic diversity of the microbivalve *Kidderia subquadrata*, reveals new data from West Antarctic Peninsula

**DOI:** 10.1038/s41598-021-85042-7

**Published:** 2021-03-11

**Authors:** Daniela Levicoy, Kamilla Flores, Sebastián Rosenfeld, Leyla Cárdenas

**Affiliations:** 1 Centro FONDAP- IDEAL, Instituto de Ciencias Ambientales and Evolutivas, Facultad de Ciencias, Universidad Austral de Chile, Independencia 641, P.O. Box 567, Valdivia, Punta Arenas, Chile; 2 Laboratorio de Ecosistemas Marinos Antárticos Y Subantárticos, Universidad de Magallanes, Casilla 113-D, Punta Arenas, Chile; 3 Laboratorio de Ecología Molecular, Departamento de Ciencias Ecológicas, Facultad de Ciencias, Universidad de Chile, Las Palmeras # 3425, Ñuñoa, Santiago Chile; 4 Instituto de Ecología y Biodiversidad (IEB), Las Palmeras # 3425, Ñuñoa, Santiago Chile

**Keywords:** Ecology, Evolution, Genetics, Ecology, Ocean sciences

## Abstract

It is well established that Antarctic biodiversity has been strongly influenced by rapid climatic fluctuations during the Quaternary. Marine invertebrates from Antarctica constitute an interesting lens through which to study the impacts of the last glacial periods as glaciation impacted the distribution and intraspecific genetic variation of these animals. However, the impact on the spatial genetic distribution and historical demography of local processes in areas adjacent to the West Antarctic Peninsula (WAP) is less clear. Here we present new genetic information on the bivalve *Kidderia subquadrata*, a small mollusk that inhabits intertidal rocky island ecosystems throughout the WAP. Using a phylogeographical approach, we examined the spatial patterns of genetic diversity in this brooder species to test the hypothesis of strong genetic structure in incubating organisms and the hypothesis of glacial refugia in organisms with limited dispersion. We found evidence of strong genetic structure among populations of the WAP and a recent expansion in the South Shetland Islands. Our findings are concordant with the predictions that incubating organisms, abundant in Antarctica, present a strong genetic structure among their populations and also support the hypothesis of glacial refugia in organisms with limited dispersion. The effect of the coastal current pattern in the WAP is suggested as a driver to the local spatial dynamics of the genetic diversity distribution. Although genetic information about this microbivalve is still scarce, the knowledge reported here has increased our understanding of the evolutionary patterns of this organism that is endemic to the Southern Ocean.

## Introduction

Studies based on Southern Ocean (SO) biodiversity have been focused on eurybathy, circumpolarity and the high prevalence of brooders^1,2^. For example, the poor dispersers, which include many species, present seemingly high levels of endemism in the SO^3^. The high prevalence of brooders can be explained partially by glacial cycles^4^. This explanation is based on the glacial refugium hypothesis^5^. In this scenario, the hypothetical glacial refuges in the Antarctic would have allowed the Antarctic species to be protected during the interglacial periods^2,6^, and processes of expansion and contraction could have taken place^7^. Currently, numerous evidence of an increase in population size during the early stages of the last glaciation seems to support the glacial contractions and interglacial expansions were a recurrent pattern that eventually led to species adapting to cold conditions during the Late Quaternary^6–10^. Species colonizing different environments have managed to survive through the Pliocene and Pleistocene glaciation cycles by shifting between habitable areas of polynyas or ice fracture zones under former or extant Antarctic ice shelves^11–13^. Some hypotheses that have emerged to explain the survival of species in the last glacial periods are derived from the Expansion‐Contraction Model^14^. One of these hypotheses is in situ persistence in Antarctic glacial refuges^11,15,16^. The in situ persistence scenario suggests the presence of one or several isolated refugia on the shelf; these refugia are associated with strong population bottlenecks^4,16^. A second hypothesis is that of island refuges, which proposes that shallow marine species survived out of the Antarctic continental shelf in adjacent Antarctic islands such as the South Shetland Islands (SSIs)^11,17^. This area has been defined as an in situ refuge associated to volcanoes or areas of geothermal activity^18^. These refuges have been particularly informative in reconstructing the periglacial and postglacial history of marine organisms^19,20^. Finding a pronounced geographical structure may provide evidence of putative refugial areas^21–23^. Refugial populations are usually composed of subsets of the genetic diversity and long-term isolation of populations within geographically separate refugia will lead to genetic differentiation^14,24^.

Despite the fact that several genera of bivalves have survived the glacial periods, a reduced number of these persist in Antarctic benthic systems and their biogeography patterns have yet to be exhaustively studied. The review of Seymour Island Paleocene molluscs^25^ lists 57 species and 41 genera, of which only 12 genera are still represented in the SO; most of the remaining genera occur at present in the seas north of the Polar Front^26^ and a few still live around Antarctica (12.5%)^25^. Moreover, the analysis of species richness in bivalve families revealed that the distribution has high richness north of the Antarctic peninsula and low richness to the south in, for example in families like Pectinidae, Nuculidae, Mytilidae, Gaimardiidae^26–35^. Antarctic bivalves have a varied range of reproductive strategies. In some the sexes are separate (gonochoric organisms) while others are simultaneous hermaphrodites^36,37^, some display indirect development and external fertilization (planktonic larvae)^38^ while others exhibit direct development in which the embryos are retained by the female, who provides parental care to the offspring (brooder species)^39–45^.

The microbivalve *K. subquadrata Pelseneer (1903)*^46,47^ is a brooder species with a 4.5 mm average shell length^47^. They incubate their offspring until the juvenile stage and have been reported inhabiting the rocky intertidal of islands adjacent to the West Antarctic Peninsula **(**WAP)^48^. No genetic information on this species has been reported prior to this work. In this paper, we aim to increase the available information about the Antarctic biodiversity and microbivalve species. To that end, we have developed a spatial analysis of the genetic diversity in populations of *K. subquadrata* from the WAP to advance the understanding of the evolutionary history of these microbivalves that inhabit Antarctica.

Based on the absence of the larval phase, we hypothesized that due to a low dispersal potential in this species we would find a high spatial genetic structure among populations with evidence of in situ refugia in the SSIs. To test this hypothesis, we used the mitochondrial molecular marker, Cytochrome oxidase I *(CoxI)* and a suite of phylogeographic analyses to estimate the spatial genetic diversity and the historical demography of this species.

## Results

### Genetic diversity and spatial genetic structure

**Figure 1:**
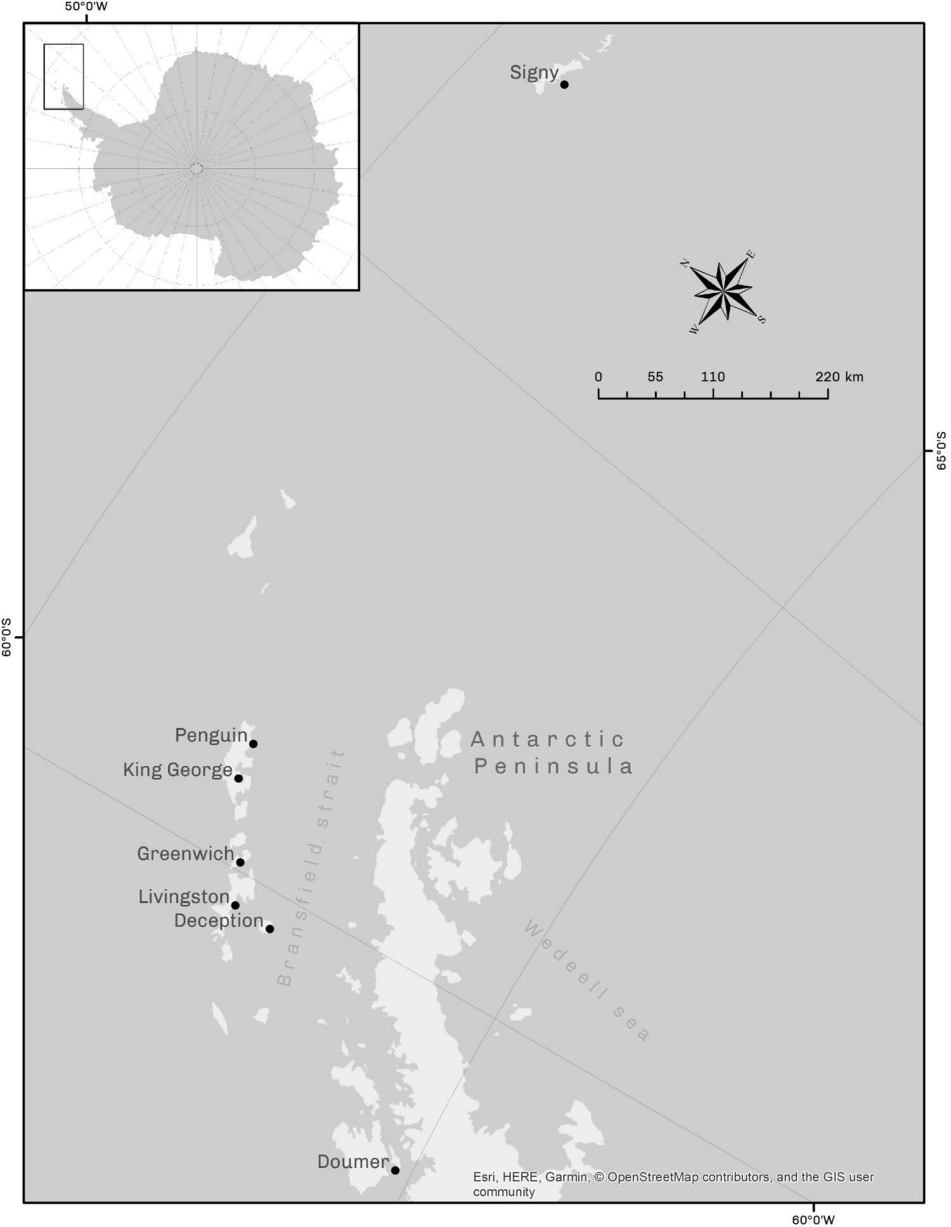
Map of West Antarctic Peninsula depicting sampling locations of *K. subquadrata*: Signy(1); Penguin(2), King George(3), Greenwich(4), Deception(5), Livingston(6), and (7) Doumer Islands.

**Figure 2:**
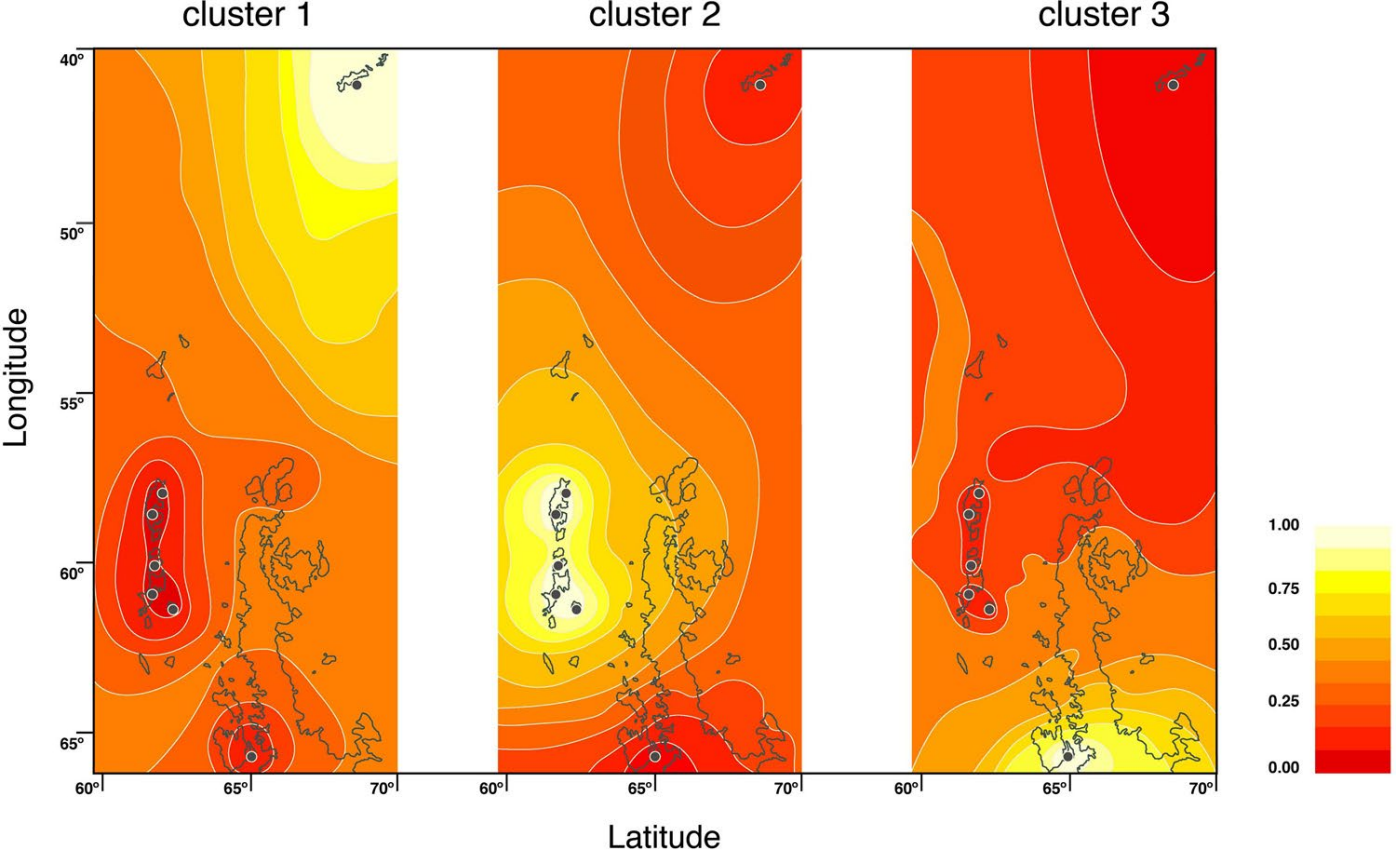
Spatial output of the Geneland analysis of *K. subquadrata* populations. Black circles indicate the relative positions of the sampled populations. Darker and lighter shading are proportional to posterior probabilities of membership in clusters, with lighter (yellow) areas showing the highest probabilities of clusters. Cluster 1: Signy Island; Cluster 2: Penguin, King George, Greenwich, Livingston and Deception Islands: Cluster 3: Doumer Island.

The study included seven localities across the WAP (Fig. 1). No insertion/deletions or stop codons were detected in the sequence dataset. In total 37 haplotypes were recovered (Table S1), with a whole dataset haplotype diversity of 0.722 ± 0.032 and 51 polymorphic sites (Table 1). The pairwise FST distance revealed a high diferentiation among sites, with Doumer island being high and significantly differentiated from the other locations (Table S1). The IBD-Mantel test indicated that the relationship between the geographic distance and the linearized genetic distance was not significant among islands (r = 0.196; p-value = 0.25). The general AMOVA showed high genetic structure among populations (F_ST_ = 0.87, p-value = 0.0001). The highest values of genetic differentiation measured with SAMOVA analysis were detected when the populations were separated into 4 groups (Group 1: Signy Island; Group 2: King George, Penguin, Greenwich, Deception Islands; Group 3: Livingston Island and Group 4: Doumer Island) with values of F_CT_ = 0.92 (p-value = 0.029) (Table 2). The model based on the Bayesian clustering algorithm and implemented in Geneland detected three clusters for the dataset (K = 3), with a high posterior probability of cluster membership (p-value = 0.9). Thus, demonstrating the strong genetic structure of *K. subquadrata* populations from the WAP. Cluster 1 (Fig. 2) includes samples exclusively from Signy Island; Cluster 2 includes samples from King George, Penguin, Greenwich, Deception and Livingston Islands; and, finally, Cluster 3 includes samples from Doumer Island.

**Table 1:** Geographic information and standard genetic statistics of *Kidderia subquadrata* sampling localities.

Locality	Coordinates	*n*	*K*	*H*	*S*	*Π*	*π*	Tajima’s D	Fu’s F_S_
Signy Island	60°43′ S–45°36′W	16	3	0.491 ± 0.117	20	9.108 ± 4.425	0.0079 ± 0.004	2.056	10.745
King George island	62°05′S–57°56′ W	32	9	0.569 ± 0.102	8	0.839 ± 0.613	0.0007 ± 0.0005	− 1.729*	− 6.132**
Penguin Island	62°05′S–57°55′W	34	7	0.373 ± 0.105	7	0.467 ± 0.419	0.0004 ± 0.0004	− 2.087**	− 5.634**
Greenwich Island	62°48′S–59°66′W	33	13	0.672 ± 0.093	15	1.265 ± 0.818	0.0011 ± 0.0008	− 2.174**	− 9.978**
Livingston Island	62°39′S–60°36′W	6	4	0.800 ± 0.172	7	2.333 ± 1.476	0.0020 ± 0.0014	− 1.390*	− 0.219
Deception island	62°58′S–60°33′W	23	4	0.439 ± 0.114	3	0.474 ± 0.428	0.0004 ± 0.0004	− 1.062	− 1.556
Doumer	64°52′S–63°35′W	35	2	0.111 ± 0.07	1	0.111 ± 0.184	0.00009 ± 0.0002	− 0.807	− 0.572
Whole dataset		179	37	0.722 ± 0.032	51	9.251 ± 4.272	0.0081 ± 0.0041	− 1.027	− 1.906

*n* = number of individuals; *k* = number of haplotypes; *S* = polymorphic sites; *H* = haplotype diversity; *Π* = mean number of pairwise differences *π* = nucleotide diversity.

p value (*) > 0.05; (**) > 0.001.

**Table 2:** Analysis of molecular variance in *K. subquadrata*.

	Tested structure	Statistics	P-values
AMOVA	(King George, Penguin, Greenwich, Deception, Livingston, Doumer)	FST = 0.87	0.000
SAMOVA			
1	(King George, Penguin, Greenwich, Deception) (Signy, Livingston, Doumer)	FSC = 0.50	0.000
		FST = 0.92	0.000
		FCT = 0.84	0.029
2	(King George, Penguin, Greenwich, Deception) (Livingston) (Signy, Doumer)	FSC = 0.35	0.000
		FST = 0.92	0.000
		FCT = 0.88	0.009
3	(King George, Penguin, Greenwich, Deception) (Signy) (Livingston) (Doumer)	FSC = 0.001	0.000
		FST = 0.92	0.000
		FCT = 0.92	0.029

Output of the AMOVA and SAMOVA analysis.

F_SC_: differentiation within islands among groups; F_CT_ differentiation among groups and Fst estimates the differentiation among islands.

**Figure 3:**
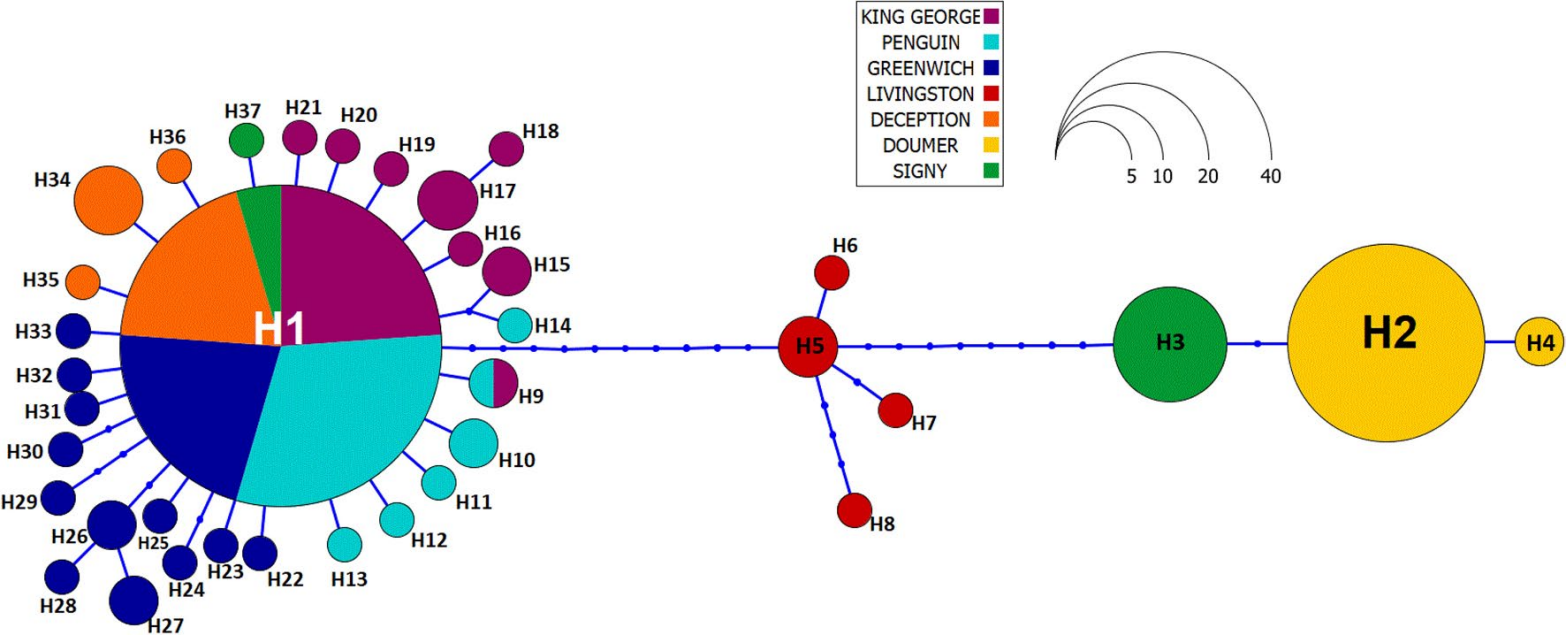
Median-joining haplotype network. Each haplotype is represented by a colored circle indicating the main area where it was collected; the size of the circle is proportional to its frequency in the overall sampling effort.

The genealogical reconstruction of the haplotype network comprised 37 different haplotypes and showed a central haplotype (H1, Fig. 3) with the highest frequency (49.6%) that is distributed among five islands (Signy, King George, Penguin, Greenwich and Deception Islands) in a star-like topology. Additionally, most of the islands presented unique haplotypes with frequencies of 0.6% (Signy), 3.4% (Deception), 3.9% (Penguin), 5.6% (King George) and 7.8% (Greenwich). Ten mutation steps separated H1 from the samples of Livingston Island, where 4 unique haplotypes (3.4% of the total haplotypes) were detected. Another 10 mutational steps separated these groups from the second most frequent haplotype (H2, 19.5%); this group include exclusively samples from Doumer Island, where 2 unique haplotypes were found. A unique haplotype from Signy Island (H3) is separated from those of Doumer Island by one mutational step (Fig. 3).

### Historical demography

**Figure 4:**
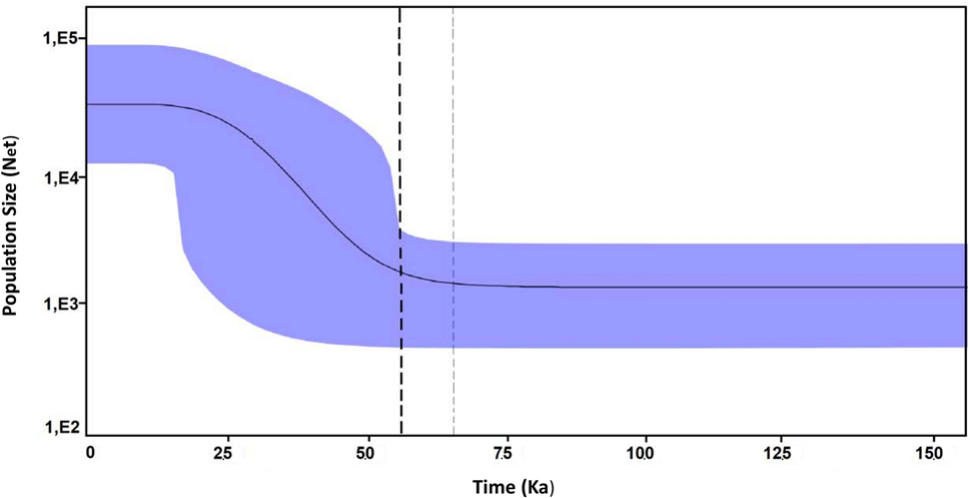
Historical demographic trends. Historical demographic trends of effective population size (Ne) constructed using a Bayesian skyline plot approach based on *CoxI* haplotypes. The y-axis is the product of effective population size (Ne) and generation length in a log scale while the x-axis is the time before present. The median estimate (solid black line) and 95% highest probability density (HPD) limits (purple area) are shown. The thick dashed line represents the time of the most recent ancestor, and the thin dashed line represents the time during which species expansion took place.

The Tajima D test and the Fu Fs statistic (Table 1) were negative but no significant for the data set as a whole (Tajima D = -1.027, p-value = 0.2; Fu´s test = -1.906, p-value = 0.2). However, both indexes were both negative and significant for most of the individual islands. Signy, Deception and Doumer Islands were the exception, with Signy island showing positive but not significant values. The Bayesian Skyline plot analysis, where Signy, Penguin, King George, Greenwich, Deception Islands were included (start-like topology in the haplotype network) supports the hypothesis of a recent population expansion of *K. subquadrata* (Fig. 4). Based on this analysis, the time of the most recent common ancestor (trmca) was 5500 years ago, while the onset of population expansion occurred approximately 65,000 years ago.

**Figure 5:**
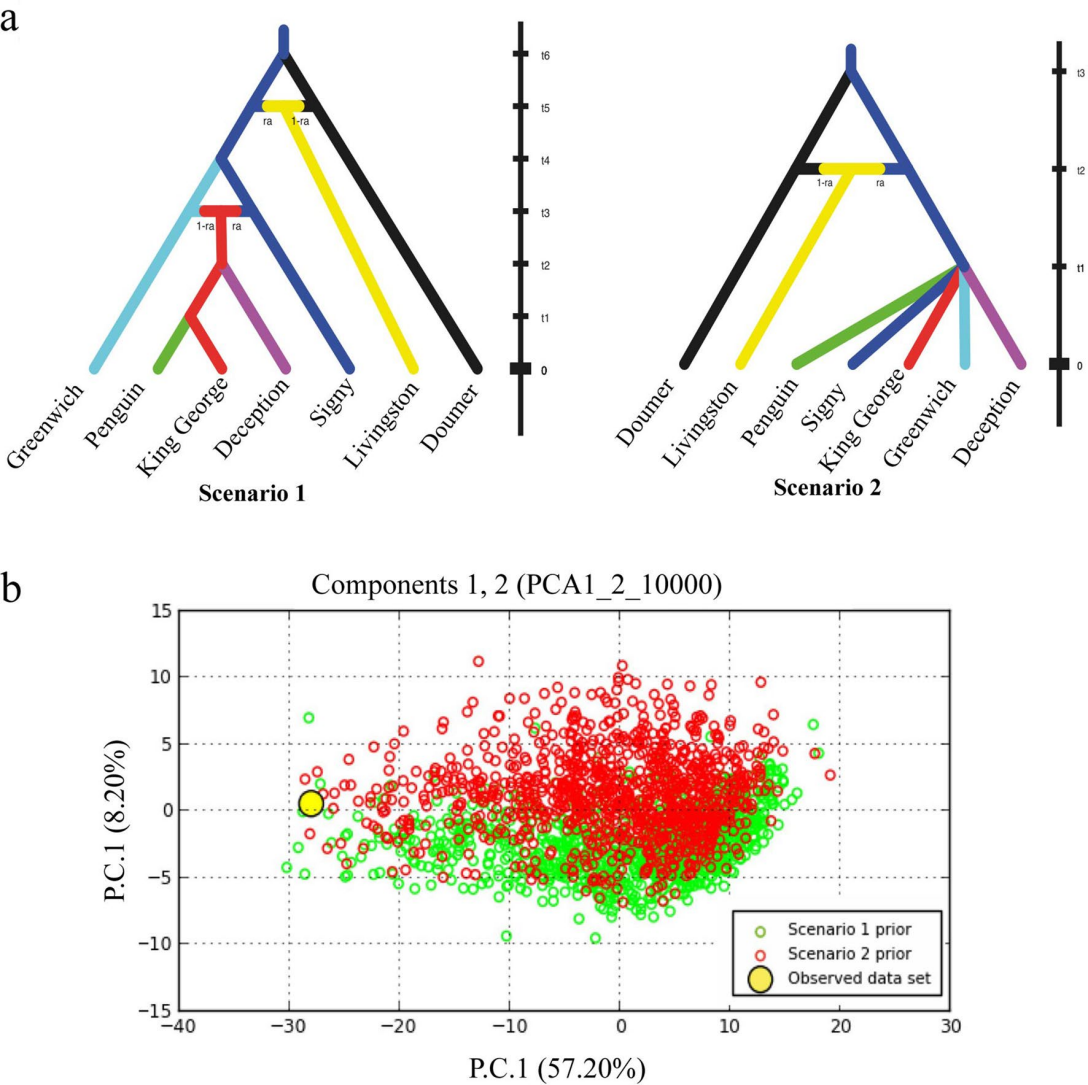
Resulting plots from DIYABC analysis. (**a**) Panel depicted the two models evaluated after the hierarchical analysis. (**b**) show the pre-evaluation step of scenarios tested, conducted through a PCA on summary statistics of simulated and observed datasets. The observed dataset (yellow dot) falls within the cloud of simulated points. See Figure S1 for details on the tested scenarios.

The pre‐evaluation step of the ABC procedure performed in DIYABC allowed to reduce the number of scenrios to test and improve DIYABC ability to reveal the true demographic model (Fig. S2–S4). The final analysis (Fig. 5) suggested both scenarios were realistic. The scenario 1 and 2 supports an initial divergence between Doumer and Signy islands and posterior admixture in which the population of Livingston island originates. The scenario 1 reveals an admixture event between Signy and Greenwich Islands giving birth to admixed populations in SSIs (King George, Penguin and Deception Islands). The scenario 2 supports a recent divergence in SSIs (Fig. 5). When scenarios were compared, posterior probabilities for the scenario 1 based on direct estimations reached the higest value of 0.8220 (0.4867,1.0000) while based en logistic approach, the scenario 2 reached the value of 0.9212 (0.9067, 0.9358). Type I error for both scenarios were low (scenario 1: Type I error = 0.038 for direct estimation; 0.009 for logistic approach; scenario 2: Type I error = 0.0039 for direct estimation; 0.010 for logistic approach).

## Discussion

Our analysis supports the genetic differentiation among sampled populations of *K. subquadrata*, in accordance with the prediction that in the WAP, this brooder species have limited dispersal potential and would therefore display a highly structured population^2,49,50^. Reproductive life history might have a crucial influence in the genetic structure patterns and consequently in the degree of connectivity among populations in Antarctic organisms. For example, a study of two common Antarctic benthic surface-grazing gastropods with contrasting development strategies revealed a high spatial structure among populations in the brooder *Margarella antarctica* while no spatial population structure was found in the broadcaster *Nacella concinna* (planktonic larva) throughout the geographical range studied^51^. Similar results were reported in other works on this Antarctic limpet; these studies, whose sampling sites covered a large geographical range, suggest the existence of a single panmictic unit^17,52^. More recently, for sea star brooders, researchers have concluded that broadcasters are less spatially structured than brooders^53^.

The local oceanographic dinamyc may have also played a relevant role in the spatial interaction among populations in the interglacial periods in the SO. The spatial genetic structure pattern of *K. subquadrata* may have been enhanced by the Bransfield Strait current system. The surface circulation in Bransfield Strait is influenced by the Antarctic Coastal Current (ACC) and the Antarctic Slope Front^54^. Seaward of the South Shetland Islands (SSIs), the southwestward slope currents persist over the long term, they are originated by the Antarctic Slope Current that flows around the Antarctic Peninsula^55^. The water masses of the ACC transport nutrients west of the SSIs and enter the area through the Bransfield Strait flowing strongly to the east-northeast along the southern margin of the SSIs^56^. The Weddell Sea water enters through the Bransfield Strait mainly from the north-east and flows in a south-west direction along the Antarctic Peninsula. A similar process has been proposed to explain a genetic barrier identified in the Antarctic annelids of the family Phyllodocidae family in the strait north of Deception Island^57^, which coincides with the oceanic front that is generated by the intrusion of seawater masses from the Weddel Sea in the Bransfield Strait^55^. Therefore, the structure in *K. subquadrata* populations could be explained by the system of currents in the Bransfield Strait that forms a cyclonic circulation^58,59^. In addition, Livingston Island showed the presence of unique haplotypes that are separated by more than 10 mutational steps from the ancestral polymorphic haplotype but lie geographically lying within the SSIs. This undoubtedly requires a more detailed explanation and further investigation. According to a modified map from Barlett (2018), Livingston Island has low bathymetry (100–200 m)^60^. This pattern could result in water retention processes that increase the retention of marine invertebrate in the island, thus acting as a sink that results in a strong spatial genetic differentiation of its *K. subquadrata* population. However, the number of individuals analyzed in this study is small and an increased sample size would provide a more complete picture of the spatial process in this locality and could reveal the actual phylogeographic pattern.

On the other hand, glacial activity in Antarctica has been suggested as a driver of phylogeographic patterns for species by causing isolation and the presence of in situ glacial refuges^2,12–14,61^. It now appears that the continental shelf was not ice-covered equally across the Antarctic coastline, which allowed some ice-free refuges for fauna during the glacial maxima^62^. According to the literature, the last glacial maximum occurred 20,000 to 17,000 years ago and West Antarctica and the adjacent islands were apparently covered in ice^62,63^. The deglaciation of Signy Island and the SSIs began 14,000—11,000 years ago and spread during the Holocene until 8,000—6,000 years ago^64–67^. Strong signals of recent demographic expansion as well as founder effect associated with recolonization can be inferred from our results in the haplotype network and DIYABC analysis. Therefore, a potential historical demographic hypothesis could be that strong genetic subdivision is a consequence of a pattern of multiple glacial refugia with a Pleistocene post-glacial expansion^19,21^.

In particular, the most widely distributed and frequents haplotypes were shared by five islands: Signy, Penguin, King George, Greenwich and Deception. The Signy and Doumer Islands are the most geographically distance localities within the study’s sampling range. The DIYABC reveals that Signy and Doumer are the ancestral populations and probably after the original split, Signy island constituted a source population (refuge) for the population of SSIs. Interestingly, the haplotype network showed that Signy also has a unique haplotype that is different from the ancestral polymorphic haplotype and more closely related to the unique Doumer haplotypes. Some individuals of *K. subquadrata* that were collected as part of our samples lived as epibionts on the macroalgae. Macroalgal rafting has been suggested to explain the low genetic differentiation of marine communities across the Subantarctic region^68^, and recent investigations have revealed the arrival of invasive species that reached the Antarctic continent alive on kelp rafts^69,70^. Thus, a potential explanation for this unusual spatial pattern of the genetic distribution in *K. subquadrata* populations could be that they have used macroalgae as a transport vector. This hypothesis implies some degree of successful colonization and posterior isolation of this unique haplotypes. In the case of Doumer Island, the southernmost locality, we identified exclusively private haplotypes*,* they displayed low genetic diversity and the highest F_ST_ values, suggesting a process known as leptokurtic dispersion^71–73^. Since that time it has probably served as its own refuge and undergone divergence in isolation^19^. Similar patterns of genetic structure have been reported in the marine bivalves *Arctica islandica*^74^ in the Northern Hemisphere.

Non‐pelagic development has been described in the Antarctic fauna, where brood protection appears dominant^75^. It is worth noting that cryptic species are commonly found in brooding species in the Antarctic Peninsula in, for example, echinoderms such as ophiuroids, echinoids and brooding pygnogonids^44,76–78^. The study of the bivalve *Lissarca notorcadensis* was one of the first to show indications of cryptic speciation in Antarcic bivalves^27^. New evidence of two cryptic lineages in the Antarctic Peninsula was recently reported in the bivalve *Aequiyoldia eightsii*, with an estimation of genetic distance of 5.79% from *CoxI*^35^. Considering *K. subquadrata’s* intrinsically low dispersal and the evidence of the probable occurrence of in situ refuges revealed in this study, a cryptic speciation process could potentially explain the genetic diversity in the population of Doumer Island. The samples obtained from Doumer Island were collected in a protected bay surrounded by glaciers, semi-isolated from the marine currents since there is only one entrance to the bay; these geographical features indicate that gene flow out of bay could be difficult. Here, based on *CoxI* we report a genetic distance of 2% among the central haplotype (H1) and the two unique haplotypes from Doumer Island; this value is within the limit established in the existing literature^79^ for inferring a speciation process. Intraspecific divergence with *CoxI* is rarely greater than 2% in the phylogeographic analyses; even so it can serve as an effective tool in recognizing *putative new lineages*^61,73,80,81^. Moreover, there are reports of distinct glacial refugia in the SSIs and Antarctic Peninsula harboring cryptic species that have diverged recently in micro-allopatry^82–84^.

In conclusion, we report new evidence of a strong spatial genetic structure in a brooder microbivalve species unique to the WAP. We also suggest possible presence of in situ glacial refuges and we infer a cryptic speciation in progress. Mitochondrial DNA has been widely used in numerous studies of phylogeography^5,16,17,21,27,35,42^; its greatest utility is in non-model species where there are no previous genetic data, since its use enables comparisons with previous studies. However, recent studies have reported significant dissimilarities between genetic diversity and structure encountered in marine species depending on the genetic markers used^85^. Differences in patterns of genetic structure have been linked to the fact that organelle DNA is more sensitive to introgression and/or rapid sweeps (due to selection or strong genetic drift) than is nuclear DNA^86^. Therefore, it is imperative to develop new nuclear markers (e.g., microsatellites or SNPs) in Antarctic marine invertebrates. Further studies could increase the number of populations sampled in the Southern Ocean to test for the existence of local adaptation using recently developed genomics and transcriptomics tools.

## Methods

### Sample sites and collection

A total of 179 individuals of *K. subquadrata* were collected from the following islands (Fig. 1): (1) Signy (60°43′ S; 45°36′W; n = 16), (2) Penguin (62°05′S- 57°55′W; n = 34), (3) King George Island (62°05′S- 57°56′ W; n = 32), (4) Greenwich (62°48′S- 59°66′W; n = 33), (5) Livingston (62°39′S- 60°36′W; n = 6), (6) Deception (62°58′S- 60°33′W; n = 23) and (7) Doumer (64°52′S-63°35′W; n = 35). In addition, we sampled twice in the Chilean Antarctic O’Higgins military base on Kopaitik Island (63°19′S; 57°53′W), but we were unable to find specimens of *K. subquadrata* in this area. All samples were collected in rocky intertidal with boulders or stones and shallow subtidal environments. The collections were performed during austral summer expeditions in 2018 and 2019.

### DNA extraction, amplification, sequencing and alignment

The total DNA of each individual was isolated with Quick—DNA plus (Zymo Research) commercial kit following the procedures described by the manufacturer. Given the absence of genetic information for the genus *Kidderia*, the strategy to generate molecular markers was to perform an Myseq Illumina sequencing using the genomic DNA and an in silico enrichment to obtain fragments of the mitochondrial genome. The total length of the partial mitochondrial genome recovered was 1733 bp. Using this genome data, specific primers for the Cytochrome oxidase I *(CoxI)* gene two pair primers were designed. Two pairs of primers were used: the first corresponds to kg1F (5′-TTG GGC TGG GTT AAT AGG TACA-3′) and kg7R (5′-GAA AAC CAG CAA ACA TAG CA-3′) flanking a fragment with a total length of 861 bp; the second pair corresponds to kg7F: (5′- TGC TAT GTT TGC TGG TTT TC -3′) and kg8R (5′- CCC AAA AAG ACA TTT GAC CC -3′), which were used to recover a fragment of 279 bp. The *CoxI* gene amplified a total length of 1140 bp, coding 380 amino acids.

All PCRs were performed in a final volume of 25 μL containing 1X PCR buffer, 3.5 mM MgCl_2_, 0.2 mM each dNTP, 0.25 μM each primer, 0.2X BSA, 1 μL DNA concentrate (10—50 ng), 0.6 units *GoTaq* DNA polymerase (Promega) and H_2_O to reach the final volume. The *CoxI* gene was amplified using the following thermocycling profile: an initial denaturation step (95 °C for 5 min); 35 cycles of amplification (94 °C for 30 s, 60 °C for 1 min and 72 °C for 2 min); and a final extension step (72 °C for 8 min). The PCR products were purified using E.Z.N.A. Cycle Pure PCR Purification kits (Omega Bio-tek) and sequenced in both directions by the sequencing service of Macrogen Inc. Company (www.macrogen.com). Sequences were aligned in Geneious R10.2.4 software^87^ and manually edited to resolve unclear base calls. *CoxI* consensus sequences were translated into amino acids using the invertebrate mitochondrial genetic code to check for stop codons. Alignments were performed using the default settings of the ClustalW alignment algorithm implemented in Geneious (cost matrix: IUB; gap open cost: 15; and gap extend cost:6.6).

### Spatial pattern of the genetic diversity

To estimate the levels of genetic polymorphism in populations of *K. subquadrata* we used standard diversity indexes: number of haplotypes (k), number of segregating sites (S), haplotype diversity (H) and the average number of paired differences between sequences (π) for each region using DnaSP v5^88^. Pairwise genetic differentiation (pairwise F_ST_) was estimated to determine the genetic structure among populations using the ARLEQUIN program v.3.51^89^. To test the hypothesis of isolation by distance (IBD) we used the relationship between genetic (FST⁄1-FST)^90^ and the geographic distances among Signy, King George, Penguin, Deception, Greenwich, Livingston and Doumer Islands to perform a Mantel test^91^ executed in the R environment with the Vegan (2.5–2 version) package^92^.

To test for spatial genetic differentiation, we calculated a global FST by performing a global AMOVA analysis with the ARLEQUIN^89^. Next, we developed a set of analyses using the geographic coordinates of each sampled island. Here, we analyzed the population structure without an a priori cluster hypothesis using a spatial analysis of molecular variance (SAMOVA)^93^. In this approach, sample sites are clustered based on a simulated procedure that aims to maximize the proportion of total genetic variance caused by differences among populations groups.

To infer the spatial pattern of genetic diversity in *K. subquadrata* we estimated the number and composition of panmictic groups and the spatial boundaries among them using a clustering method based on the Bayesian model computed with the GENELAND package, version 4.0.0^94^ in the R environment version 2.4.1^92^. This software implements a Markov Chain Monte Carlo (MCMC) procedure to determine the best clustering of samples with regard to genetic and geographic information. Geographic information is considered as the Bayesian prior level, so clusters corresponding to spatially structured groups are considered to be more probable than clusters that are randomly distributed in space. Five × 10^6^ MCMC iterations were sampled every 1000 steps with a 500-step burn-in period, and a maximum number of clusters K = 8 was run to estimate the model parameters and posterior probabilities of group membership.

Finally, the genealogical relationships among *CoxI* haplotypes for the whole dataset were characterized using median joining networks in HapView (http://www.cibiv.at/~greg/haploviewer)^95^.

### Historical demography

To examine the historical population demography, the Tajima D test^96^ and the Fu Fs statistic^97^ were performed. The Tajima D test is based on the fact that in a neutral model, estimates of the number of sites segregating and the average number of nucleotide differences are correlated. Fu’s test is based on the model of infinite sites without recombination; it gives the probability of observing a random sample with the number of alleles equal to or less than the value observed, given the observed level of diversity and the assumption that all alleles are selectively neutral. Both statistics were calculated in the ARLEQUIN. In addition, we estimated past population dynamics over time using the Bayesian skyline plot method implemented in BEAST 2^98^. To develop this analysis, we selected the five sample sites (Signy, Penguin, King George, Greenwich, Deception Islands) that shared the most frequently observed haplotype; as described earlier, these sample sites showed a star-like topology in the haplotype network. We conducted two independent Bayesian MCMC runs using the generalized time-reversible (GTR) model with a gamma distribution (G), previously estimated with JModelTest2^99^ and mutation rates calibrated for *CoxI* sequences of bivalves^100,101^ 1.0% Myr^-1^. Substitution rates were modified to a tenfold evolutionary rate (10% per million), considering the correction for time dependence of molecular rates at the population level^102,103^. The two independent MCMC calculations were run for 1.5 × 10^7^ generations (sampled every 1000 iterations), and the first 10% of the trees were discarded as burn-in. The convergence of runs was confirmed with Tracer v1.6^104^, ensuring a minimum of 600 effective samples for each statistic (ESSs). The median and corresponding credibility intervals of the Bayesian skyline plot were depicted with Tracer v1. 6.

In order to better understand the population history of the species we used the program DIYABC v2.1^105^ to tested for different population demographic scenarios. This software evaluates population histories using Approximate Bayesian Computation (ABC) with genetic data, by testing scenarios that are built through a combination of population divergence, admixture and population size changes. Following the recommendations of Cabrera and Palsbøll^106^ to improve DIYABC’s ability to reveal the true demographic model, we focused on simple contrasting models and reduced the number of candidate scenarios after a set of hierarchical analysis beginig with 5 preliminary scenarios (Fig. S1). Subsequently, and following the results of the previous genetic analysis, two models were evaluated at the last level (Fig. 5). The mutation rate was set with a mínimum of 1 × 10^–9^ and máximum 1 × 10^–4^ and the mutation model used was kimura 2 parameters. For the historical models, priors were set by default and in accordance with the recommendations of the authors of the software, we performed 2,000,000 simulations.

## Supplementary information

Supplementary information.

## Data Availability

The sequences of dataset were deposited in Genbank; accession IDs: MT712476—MT712654.

## References

[1] Griffiths HJ, Barnes DK, Linse K (2009). Towards a generalized biogeography of the Southern Ocean benthos. J. Biogeogr..

[2] Halanych KM, Mahon AR (2018). Challenging dogma concerning biogeographic patterns of Antarctica and the Southern Ocean. Annu. Rev. Ecol. Evol. Syst..

[3] Broyer, C. & Koubbi, P. Chapter 1.1. Phylogeography and population genetics. In: De Broyer C., Koubbi P., Griffiths H.J., Raymond B., Udekem d’Acoz C. d’, *et al*. (eds.) *Biogeographic Atlas of the Southern Ocean* (Scientific Committee on Antarctic Research, Cambridge, 2014) pp. 2–5.

[4] Allcock AL, Strugnell JM (2012). Southern Ocean diversity: new paradigms from molecular ecology. Trends Ecol. Evol..

[5] Holder K, Montgomerie R, Friesen VL (1999). A test of the glacial refugium hypothesis using patterns of mitochondrial and nuclear DNA sequence variation in rock ptarmigan (*Lagopus mutus*). Evolution.

[6] Stewart JR, Lister AM, Barnes I, Dalén L (2010). Refuges revisited: individualistic responses of species in space and time. Proc. R. Soc. Lond. B Biol. Sci..

[7] Hewitt GM (1996). Some genetic consequences of ice ages, and their role in divergence and speciation. Biol. J. Lin. Soc..

[8] Hewitt GM (2004). The structure of biodiversity–insights from molecular phylogeography. Front. Zool..

[9] Fedorov VB, Stenseth NC (2002). Multiple glacial refugia in the North American Arctic: inference from phylogeography of the collared lemming (*Dicrostonyx groenlandicus*). Proc. R. Soc. Lond. B Biol. Sci..

[10] Dalén L, Nyström V, Valdiosera C, Germonpré M, Sablin M, Turner E, Götherström A (2007). Ancient DNA reveals lack of postglacial habitat tracking in the arctic fox. Proc. Natl. Acad. Sci..

[11] Thatje S, Hillenbrand CD, Mackensen A, Larter R (2008). Life hung by a thread: endurance of Antarctic fauna in glacial periods. Ecology.

[12] Weihe E, Abele D (2008). Differences in the physiological response of inter-and subtidal Antarctic limpets *Nacella concinna* to aerial exposure. Aquat. Biol..

[13] Abele D, Vazquez S, Buma AG, Hernandez E, Quiroga C, Held C, Mac Cormack WP (2017). Pelagic and benthic communities of the Antarctic ecosystem of Potter Cove: genomics and ecological implications. Mar. Genom..

[14] Provan J, Bennett KD (2008). Phylogeographic insights into cryptic glacial refuges. Trends Ecol. Evol..

[15] Thatje S, Hillenbrand CD, Larter R (2005). On the origin of Antarctic marine benthic community structure. Trends Ecol. Evol..

[16] Díaz A, Gérard K, González-Wevar C, Maturana C, Féral JP, David B, Poulin E (2018). Genetic structure and demographic inference of the regular sea urchin *Sterechinus neumayeri* (Meissner, 1900) in the Southern Ocean: the role of the last glaciation. PLoS ONE.

[17] González-Wevar CA, Saucède T, Morley SA, Chown SL, Poulin E (2013). Extinction and recolonization of maritime Antarctica in the limpet *Nacella concinna* (Strebel, 1908) during the last glacial cycle: toward a model of Quaternary biogeography in shallow Antarctic invertebrates. Mol. Ecol..

[18] Fraser CI, Terauds A, Smellie J, Convey P, Chown SL (2014). Geothermal activity helps life survive glacial cycles. Proc. Natl. Acad. Sci..

[19] Maggs CA, Castilho R, Foltz D, Henzler C, Jolly MT, Kelly J, Viard F (2008). Evaluating signatures of glacial refugia for North Atlantic benthic marine taxa. Ecology.

[20] Lee JR, Raymond B, Bracegirdle TJ, Chades I, Fuller RA, Shaw JD, Terauds A (2017). Climate change drives expansion of Antarctic ice-free habitat. Nature.

[21] Mcgaughran A, Torricelli G, Carapelli A, Frati F, Stevens MI, Convey P, Hogg ID (2010). Contrasting phylogeographical patterns for springtails reflect different evolutionary histories between the Antarctic Peninsula and Continental Antarctica. J. Biogeogr..

[22] Mcgaughran A, Stevens MI, Hogg ID, Carapelli A (2011). Extreme glacial legacies: a synthesis of the Antarctic springtail phylogeographic record. Insects.

[23] Fraser CI, Nikula R, Ruzzante DE, Waters JM (2012). Poleward bound: biological impacts of Southern Hemisphere glaciation. Trends Ecol. Evol..

[24] Moore JM, Carvajal JI, Rouse GW, Wilson NG (2018). The Antarctic circumpolar current isolates and connects: structured circumpolarity in the sea star Glabraster Antarctica. Ecol. Evol..

[25] Beu AG (2009). Before the ice: biogeography of Antarctic Paleogene molluscan faunas. Palaeogeogr. Palaeoclimatol. Palaeoecol..

[26] Linse, K. Chapter 5.11. Bivalvia. In: De Broyer C., Koubbi P., Griffiths H.J., Raymond B., Udekem d’Acoz C. d’, *et al*. (eds.) *Biogeographic Atlas of the Southern Ocean* (Scientific Committee on Antarctic Research, Cambridge, 2014), pp. 126–127.

[27] Linse K, Cope T, Lörz AN, Sands C (2007). Is the Scotia Sea a centre of Antarctic marine diversification? Some evidence of cryptic speciation in the circum-Antarctic bivalve *Lissarca notorcadensis* (Arcoidea: Philobryidae). Polar Biol..

[28] Arntz, W. E., Gutt, J. & Klages, M. (1997). Antarctic marine biodiversity: an overview. In *Antarctic Communities: Species, Structure and Survival* 3–14 (Cambridge University Press, Cambridge, 1997).

[29] Brandt A, Linse K, Mühlenhardt-Siegel U (1999). Biogeography of Crustacea and Mollusca of the SubAntarctic and Antarctic regions. Sci. Mar..

[30] Brandt A, Linse K, Schüller M (2009). Bathymetric distribution patterns of Southern Ocean macrofaunal taxa: Bivalvia, Gastropoda, Isopoda and Polychaeta. Deep Sea Res. Part I.

[31] Canapa A, Barucca M, Caputo V, Marinelli A, Cerioni PN, Olmo E, Odierna G (2000). A molecular analysis of the systematics of three Antarctic bivalves. Ital. J. Zool..

[32] Page TJ, Linse K (2002). More evidence of speciation and dispersal across the Antarctic Polar Front through molecular systematics of Southern Ocean Limatula (Bivalvia: Limidae). Polar Biol..

[33] Passos FD, Magalhães FT (2011). A comparative study of the Bivalvia (Mollusca) from the continental shelves of Antarctica and Brazil. Biota. Neotrop..

[34] Jackson JA, Linse K, Whittle R, Griffiths HJ (2015). The evolutionary origins of the Southern Ocean philobryid bivalves: hidden biodiversity, ancient persistence. PLoS ONE.

[35] González-Wevar CA, Gérard K, Rosenfeld S, Saucède T, Naretto J, Díaz A, Poulin E (2019). Cryptic speciation in Southern Ocean *Aequiyoldia eightsii* (Jay, 1839): Mio-Pliocene trans-Drake Passage separation and diversification. Prog. Oceanogr..

[36] Powell DK, Tyler PA, Peck LS (2001). Effect of sperm concentration and sperm ageing on fertilisation success in the Antarctic soft-shelled clam *Laternula elliptica* and the Antarctic limpet *Nacella concinna*. Mar. Ecol. Prog. Ser..

[37] Taylor JD, Glover EA, Harper EM, Crame JA, Ikebe C, Williams ST (2018). Left in the cold? Evolutionary origin of *Laternula elliptica*, a keystone bivalve species of Antarctic benthos. Biol. J. Lin. Soc..

[38] Lau, S. C., Grange, L. J., Peck, L. S. & Reed, A. J. The reproductive ecology of the Antarctic bivalve *Aequiyoldia eightsii* (Protobranchia: Sareptidae) follows neither Antarctic nor taxonomic patterns. *Pol. Biol.*, 1–14 (2018).

[39] Helmuth B, Veit RR, Holberton R (1994). Long-distance dispersal of a subAntarctic brooding bivalve (*Gaimardia trapesina*) by kelp-rafting. Mar. Biol..

[40] Hunter RL, Halanych KM (2008). Evaluating connectivity in the brooding brittle star *Astrotoma agassizii* across the Drake Passage in the Southern Ocean. J. Hered..

[41] Sherman CDH, Hunt A, Ayre DJ (2008). Is life history a barrier to dispersal? Contrasting patterns of genetic differentiation along an oceanographically complex coast. Biol. J. Linn. Soc..

[42] Thornhill DJ, Mahon AR, Norenburg JL, Halanych KM (2008). Open-ocean barriers to dispersal: a test case with the Antarctic Polar Front and the ribbon worm *Parborlasia corrugatus* (Nemertea: Lineidae). Mol. Ecol..

[43] Janosik AM, Mahon AR, Halanych KM (2011). Evolutionary history of Southern Ocean Odontaster sea star species (Odontasteridae; Asteroidea). Polar Biol..

[44] Thatje, S. Effects of capability for dispersal on the evolution of diversity in Antarctic benthos. *Integr. Comp. Biol.* ics105 (2012). 10.1093/icb/ics105 22821584

[45] Hüne M, González-Wevar C, Poulin E, Mansilla A, Fernández DA, Barrera-Oro E (2015). Low level of genetic divergence between Harpagifer fish species (Perciformes: Notothenioidei) suggests a Quaternary colonization of Patagonia from the Antarctic Peninsula. Polar Biol..

[46] Pelseneer, P. Mollusques (Amphineures, Gastropodes et Lamellibranches) Expédition Antartique Belge: Résultats Voyage du S. Y. Belgica en 1897–1898–1899 7 85, pp., pls. 1–9 (1903).

[47] Shabica, S. V. Reproductive biology of the brooding Antarctic lamellibranch *Kidderia Subquadrata* Pelseneer 1974. MSc Thesis. School of Oceanography, Oregon.

[48] Linse K, Griffiths HJ, Barnes DK, Clarke A (2006). Biodiversity and biogeography of Antarctic and sub-Antarctic mollusca. Deep Sea Res. Part II.

[49] Baird HP, Miller KJ, Stark JS (2012). Genetic population structure in the Antarctic benthos: insights from the widespread amphipod *Orchomenella franklini*. PloS one.

[50] Havermans C, Sonet G, d’Acoz CDU, Nagy ZT, Martin P, Brix S, Held C (2013). Genetic and morphological divergences in the cosmopolitan deep-sea amphipod *Eurythenes gryllus* reveal a diverse abyss and a bipolar species. PLoS ONE.

[51] Hoffman JI, Clarke A, Linse K, Peck LS (2011). Effects of brooding and broadcasting reproductive modes on the population genetic structure of two Antarctic gastropod molluscs. Mar. Biol..

[52] González-Wevar CA, Nakano T, Cañete JI, Poulin E (2010). Molecular phylogeny and historical biogeography of Nacella (Patellogastropoda: Nacellidae) in the Southern Ocean. Mol. Phylogenet. Evol..

[53] Moreau C, Danis B, Jossart Q, Eléaume M, Sands C, Achaz G, Saucède T (2019). Is reproductive strategy a key factor in understanding the evolutionary history of Southern Ocean Asteroidea (Echinodermata)?. Ecol. Evol..

[54] Thompson AF, Heywood KJ, Thorpe SE, Renner AH, Trasviña A (2009). Surface circulation at the tip of the Antarctic Peninsula from drifters. J. Phys. Oceanogr..

[55] Du G, Zhang Z, Zhou M, Zhu Y, Zhong Y (2018). The upper 1000-m slope currents North of the South Shetland Islands and Elephant Island based on ship cruise observations. J. Ocean Univ. China.

[56] Hofmann EE, Klinck JM, Lascara CM, Smith DA (1996). Water mass distribution and circulation west of the Antarctic Peninsula and including Bransfield Strait. Found. Ecol. Res. West Antarctic Peninsula.

[57] Leiva C, Riesgo A, Avila C, Rouse GW, Taboada S (2018). Population structure and phylogenetic relationships of a new shallow-water Antarctic phyllodocid annelid. Zoolog. Scr..

[58] Savidge DK, Amft JA (2009). Circulation on the West Antarctic Peninsula derived from 6 years of shipboard ADCP transects. Deep Sea Res. Part I.

[59] Veit-Köhler G, Durst S, Schuckenbrock J, Hauquier F, Suja LD, Dorschel B, Arbizu PM (2018). Oceanographic and topographic conditions structure benthic meiofauna communities in the Weddell Sea, Bransfield Strait and Drake Passage (Antarctic). Prog. Oceanogr..

[60] Barlett EMR, Tosonotto GV, Piola AR, Sierra ME, Mata MM (2018). On the temporal variability of intermediate and deep waters in the Western Basin of the Bransfield Strait. Deep Sea Res. Part II.

[61] Riesgo A, Taboada S, Avila C (2015). Evolutionary patterns in Antarctic marine invertebrates: an update on molecular studies. Mar. Genom..

[62] Sugden DE, Clapperton CM (1977). The maximum ice extent on island groups in the Scotia Sea Antarctica. Quatern. Res..

[63] Gersonde R, Crosta X, Abelmann A, Armand L (2005). Sea-surface temperature and sea ice distribution of the Southern Ocean at the EPILOG Last Glacial Maximum-a circum-Antarctic view based on siliceous microfossil records. Quatern. Sci. Rev..

[64] Ingólfsson Ó, Hjort C (2002). Glacial history of the Antarctic Peninsula since the Last Glacial Maximum—a synthesis. Polar Res..

[65] Simms AR, Milliken KT, Anderson JB, Wellner JS (2011). The marine record of deglaciation of the South Shetland Islands, Antarctica since the Last Glacial Maximum. Quatern. Sci. Rev..

[66] Herron MJ, Anderson JB (1990). Late quaternary glacial history of the South Orkney Plateau Antarctica. Quatern. Res..

[67] Anderson JB, Shipp SS, Lowe AL, Wellner JS, Mosola AB (2002). The Antarctic ice sheet during the last glacial maximum and its subsequent retreat history: a review. Quatern. Sci. Rev..

[68] Nikula R, Fraser CI, Spencer HG, Waters JM (2010). Circumpolar dispersal by rafting in two subantarctic kelp-dwelling crustaceans. Mar. Ecol. Prog. Ser..

[69] Fraser, C. I., Morrison, A. & Rojas, P. O. (2020). Biogeographic Processes Influencing Antarctic and sub-Antarctic Seaweeds. In *Antarctic Seaweeds* 43–57 (Springer, Cham, 2020).

[70] Ávila C, Angulo-Preckler C, Martín-Martín RP, Figuerola B, Griffiths HJ, Waller CL (2020). Invasive marine species discovered on non-native kelp rafts in the warmest Antarctic island. Sci. Rep..

[71] Takahata N, Slatkin M (1986). Private alleles in a partially isolated population II: distribution of persistence time and probability of emigration. Theor. Popul. Biol..

[72] Stevens MI, Hogg ID (2003). Long-term isolation and recent range expansion from glacial refugia revealed for the endemic springtail Gomphiocephalus hodgsoni from Victoria Land Antarctica. Mol. Ecol..

[73] Rogers AD (2007). Evolution and biodiversity of Antarctic organisms: a molecular perspective. Philos. Trans. R. Soc. B Biol. Sci..

[74] Dahlgren TG, Weinberg JR, Halanych KM (2000). Phylogeography of the ocean quahog (Arctica islandica): influences of paleoclimate on genetic diversity and species range. Mar. Biol..

[75] Thorson G (1950). Reproductive and larval ecology of marine bottom invertebrates. Biol. Rev..

[76] Peck LS, Bullough LW (1993). Growth and population structure in the infaunal bivalve Yoldia eightsi in relation to iceberg activity at Signy Island Antarctica. Mar. Biol..

[77] Waller CL, Barnes DK, Convey P (2006). Ecological contrasts across an Antarctic land–sea interface. Austral Ecol..

[78] Barnes DK, Conlan KE (2007). Disturbance, colonization and development of Antarctic benthic communities. Philos. Trans. R. Soc. Lond. B Biol. Sci..

[79] Hebert PD, Ratnasingham S, de Waard JR (2003). Barcoding animal life: cytochrome c oxidase subunit 1 divergences among closely related species. Proc. R. Soc. Lond. B Biol. Sci..

[80] Cook LG, Edwards RD, Crisp MD, Hardy NB (2010). Need morphology always be required for new species descriptions?. Invertebr. Syst..

[81] Chown SL, Clarke A, Fraser CI, Cary SC, Moon KL, McGeoch MA (2015). The changing form of Antarctic biodiversity. Nature.

[82] Wilson NG, Schrodl M, Halanych KM (2009). Ocean barriers and glaciation: evidence for explosive radiation of mitochondrial lineages in the Antarctic sea slug Doris kerguelenensis (Mollusca, Nudibranchia). Mol. Ecol..

[83] Allcock AL, Barratt I, Eleaume M, Linse K, Norman MD, Smith PJ, Steinke D, Stevens DW, Strugnell JM (2011). Cryptic speciation and the circumpolarity debate: a case study on endemic Southern Ocean octopuses using the COI barcode of life. Deep Sea Res. II.

[84] Verheye ML, Backeljau T, d’Acoz CDU (2016). Looking beneath the tip of the iceberg: diversification of the genus Epimeria on the Antarctic shelf (Crustacea, Amphipoda). Polar Biol.

[85] Durand JD, Blel H, Shen KN, Koutrakis ET, Guinand B (2013). Population genetic structure of *Mugil cephalus* in the Mediterranean and Black Seas: a single mitochondrial clade and many nuclear barriers. Mar. Ecol. Prog. Ser..

[86] Bonnet T, Leblois R, Rousset F, Crochet PA (2017). A reassessment of explanations for discordant introgressions of mitochondrial and nuclear genomes. Evolution.

[87] Kearse M, Moir R, Wilson A, Stones-Havas S, Cheung M, Sturrock S, Thierer T (2012). Geneious basic: an integrated and extendable desktop software platform for the organization and analysis of sequence data. Bioinformatics.

[88] Librado P, Rozas J (2009). DnaSP v5: a software for comprehensive analysis of DNA polymorphism data. Bioinformatics.

[89] Excoffier L, Lischer HE (2010). Arlequin suite ver 35: a new series of programs to perform population genetics analyses under Linux and Windows. Mol. Ecol. Resour..

[90] Rousset F (1997). Genetic differentiation and estimation of gene flow from F-statistics under isolation by distance. Genetics.

[91] Mantel N (1967). The detection of disease clustering and a generalized regression approach. Can. Res..

[92] Ihaka R, Gentleman R (1996). R: a language for data analysis and graphics. J. Comput. Graph Stat..

[93] Dupanloup I, Schneider S, Excoffier L (2002). A simulated annealing approach to define the genetic structure of populations. Mol. Ecol..

[94] Guillot G, Mortier F, Estoup A (2005). GENELAND: a computer package for landscape genetics. Mol. Ecol. Notes.

[95] Salzburger W, Ewing GB, Von Haeseler A (2011). The performance of phylogenetic algorithms in estimating haplotype genealogies with migration. Mol. Ecol..

[96] Tajima F (1989). Statistical method for testing the neutral mutation hypothesis by DNA polymorphism. Genetics.

[97] Fu YX (1997). Statistical tests of neutrality of mutations against population growth, hitchhiking and background selection. Genetics.

[98] Bouckaert R, Heled J, Kühnert D, Vaughan T, Wu CH, Xie D, Drummond AJ (2014). BEAST 2: a software platform for Bayesian evolutionary analysis. PLoS Comput. Biol..

[99] Darriba D, Taboada GL, Doallo R, Posada D (2012). jModelTest 2: more models, new heuristics and parallel computing. Nat. Methods.

[100] Marko PB, Moran AL (2009). Out of sight, out of mind: high cryptic diversity obscures the identities and histories of geminate species in the marine bivalve subgenus Acar. J. Biogeogr..

[101] Wilke T, Schultheiß R, Albrecht C (2009). As time goes by: a simple fool's guide to molecular clock approaches in invertebrates. Am. Malacol. Bull..

[102] Ho SY, Phillips MJ, Cooper A, Drummond AJ (2005). Time dependency of molecular rate estimates and systematic overestimation of recent divergence times. Mol. Biol. Evol..

[103] Hoareau TB (2015). Late glacial demographic expansion motivates a clock overhaul for population genetics. Syst. Biol..

[104] Rambaut, A., Suchard, M. A., Xie, D. & Drummond, A. J. Tracer v1 **6**, 2014 (2016).

[105] Cornuet JM (2014). DIYABC v2.0: a software to make approximate Bayesian computation inferences about population history using single nucleotide polymorphism, DNA sequence and microsatellite data. Bioinformatics.

[106] Cabrera AA, Palsbøll PJ (2017). Inferring past demographi changes from contemporary genetic data: a simulation-based evaluation of the ABC methods implemented in DIYABC. Mol. Ecol. Resour..

