# Supplementary material for: Phylogeography and genetic diversity of the microbivalve Kidderia subquadrata, reveals new data from West Antarctic Peninsula
Source: Sci Rep. 2021 Mar 11;11:5705. doi: 10.1038/s41598-021-85042-7 (PMC7952419; doi:10.1038/s41598-021-85042-7)
Supplement: Supplementary file 1 — Supplementary information. [file 41598_2021_85042_MOESM1_ESM.docx]

**Phylogeography and genetic diversity of the microbivalve *Kidderia subquadrata*, reveals new data from West Antarctic Peninsula.**

Daniela Levicoy^1^, Kamilla Flores^1^, Sebastián Rosenfeld^2^, Leyla Cárdenas^1*^

^1^ Centro FONDAP- IDEAL, Instituto de Ciencias Ambientales & Evolutivas, Facultad de Ciencias, Universidad Austral de Chile, Independencia 641, P.O. Box 567, Valdivia, Punta Arenas, Chile, ^2^ Laboratorio de Ecosistemas Marinos Antárticos y Subantárticos, Universidad de Magallanes, Casilla 113-D, Punta Arenas, Chile

*email: leylacardenas@uach.cl

**Table S1**. Haplotype distribution. Number show the individuals samples belongs to a specific haplotype by locality.

|  | Signy | Penguin | King George | Greenwich | Deception | Livingston | Doumer | **Total** |
| --- | --- | --- | --- | --- | --- | --- | --- | --- |
| Haplotype 1 | 4 | 27 | 21 | 19 | 17 | 0 | 0 | **88** |
| Haplotype 2 | 0 | 0 | 0 | 0 | 0 | 0 | 33 | **33** |
| Haplotype 3 | 11 | 0 | 0 | 0 | 0 | 0 | 0 | **11** |
| Haplotype 4 | 0 | 0 | 0 | 0 | 0 | 0 | 1 | **1** |
| Haplotype 5 | 0 | 0 | 0 | 0 | 0 | 3 | 0 | **3** |
| Haplotype 6 | 0 | 0 | 0 | 0 | 0 | 1 | 0 | **1** |
| Haplotype 7 | 0 | 0 | 0 | 0 | 0 | 1 | 0 | **1** |
| Haplotype 8 | 0 | 0 | 0 | 0 | 0 | 1 | 0 | **1** |
| Haplotype 9 | 0 | 1 | 1 | 0 | 0 | 0 | 0 | **2** |
| Haplotype 10 | 0 | 2 | 0 | 0 | 0 | 0 | 0 | **2** |
| Haplotype 11 | 0 | 1 | 0 | 0 | 0 | 0 | 0 | **1** |
| Haplotype 12 | 0 | 1 | 0 | 0 | 0 | 0 | 0 | **1** |
| Haplotype 13 | 0 | 1 | 0 | 0 | 0 | 0 | 0 | **1** |
| Haplotype 14 | 0 | 1 | 0 | 0 | 0 | 0 | 0 | **1** |
| Haplotype 15 | 0 | 0 | 2 | 0 | 0 | 0 | 0 | **2** |
| Haplotype 16 | 0 | 0 | 1 | 0 | 0 | 0 | 0 | **1** |
| Haplotype 17 | 0 | 0 | 3 | 0 | 0 | 0 | 0 | **3** |
| Haplotype 18 | 0 | 0 | 1 | 0 | 0 | 0 | 0 | **1** |
| Haplotype 19 | 0 | 0 | 1 | 0 | 0 | 0 | 0 | **1** |
| Haplotype 20 | 0 | 0 | 1 | 0 | 0 | 0 | 0 | **1** |
| Haplotype 21 | 0 | 0 | 1 | 0 | 0 | 0 | 0 | **1** |
| Haplotype 22 | 0 | 0 | 0 | 1 | 0 | 0 | 0 | **1** |
| Haplotype 23 | 0 | 0 | 0 | 1 | 0 | 0 | 0 | **1** |
| Haplotype 24 | 0 | 0 | 0 | 1 | 0 | 0 | 0 | **1** |
| Haplotype 25 | 0 | 0 | 0 | 1 | 0 | 0 | 0 | **1** |
| Haplotype 26 | 0 | 0 | 0 | 2 | 0 | 0 | 0 | **2** |
| Haplotype 27 | 0 | 0 | 0 | 2 | 0 | 0 | 0 | **2** |
| Haplotype 28 | 0 | 0 | 0 | 1 | 0 | 0 | 0 | **1** |
| Haplotype 29 | 0 | 0 | 0 | 1 | 0 | 0 | 0 | **1** |
| Haplotype 30 | 0 | 0 | 0 | 1 | 0 | 0 | 0 | **1** |
| Haplotype 31 | 0 | 0 | 0 | 1 | 0 | 0 | 0 | **1** |
| Haplotype 32 | 0 | 0 | 0 | 1 | 0 | 0 | 0 | **1** |
| Haplotype 33 | 0 | 0 | 0 | 1 | 0 | 0 | 0 | **1** |
| Haplotype 34 | 0 | 0 | 0 | 0 | 4 | 0 | 0 | **4** |
| Haplotype 35 | 0 | 0 | 0 | 0 | 1 | 0 | 0 | **1** |
| Haplotype 36 | 0 | 0 | 0 | 0 | 1 | 0 | 0 | **1** |
| Haplotype 37 | 2 | 0 | 0 | 0 | 0 | 0 | 0 | **2** |
| Total samples | | | | | | | | **179** |

**Table S2.** Pairwise FST values (bellow diagonal) and p values (Above diagonal) for each pair of sampled islands. Bold denote Non-significant comparisons.

|  | Signy | Deception | King George | Livingston | Penguin | Greenwich | Doumer |
| --- | --- | --- | --- | --- | --- | --- | --- |
| Signy |  | 0.0000 | 0.0000 | 0.0001 | 0.0000 | 0.0000 | 0.0000 |
| Deception | 0.702 |  | 0.032 | 0.000 | 0.0299 | **0.0951** | 0.0000 |
| King George | 0.723 | 0.055 |  | 0.000 | **0.0549** | 0.0394 | 0.0000 |
| Livingston | 0.360 | 0.931 | 0.912 |  | 0.000 | 0.0012 | 0.0000 |
| Penguin | 0.748 | 0.052 | 0.022 | 0.934 |  | 0.0100 | 0.0000 |
| Greenwich | 0.711 | **0.043** | **0.047** | 0.887 | 0.040 |  | 0.0000 |
| Doumer | 0.567 | 0.987 | 0.978 | 0.964 | 0.988 | 0.970 |  |

Figure S1. Graphic representation of the hierarchical ABC analysis, to compare various evolutionary history and divergence scenarios generated and tested using the program DIY-ABC. Relative posterior probabilities (Pp), and 95% confidence interval are shown for each scenario. obtained through a logistic regression computed every 10% of the number of 30,000 simulated data closest to the observed data. Rd box identified the scenarios tested in the following level.

**Figure S2:** Inference of population history and estimation of divergence using hierarchical ABC in program DIY-ABC (Level 1). A. Drawing of tested scenarios. B. Principal components analysis in pre-evaluation of scenarios. C. Compute posterior probabilities (direct approach) D. Compute posterior probabilities (logistic approach). Numerical values of C - D graphics are shown below. The scenarios with major probabilities were marked to test in the next level

Figure S3. Inference of population history and estimation of divergence using hierarchical ABC in program DIY-ABC (Level 2).

.

Figure S4. Inference of population history and estimation of divergence using hierarchical ABC in program DIY-ABC (Level 3).
